# Enhanced Creatinine Level in Diabetic Patients Maximizing the Possibilities of Nephropathy and Its Association With Blood Urea Nitrogen and Glomerular Filtration Rate

**DOI:** 10.7759/cureus.70482

**Published:** 2024-09-30

**Authors:** Batool Butt, Bushra Ghulam, Zahira Bashir, Sajid Rafique Abbasi, Shoukat Hussain, Sarosh Khan Jadoon, Amna Akbar, Mumtaz Ahmad Khan

**Affiliations:** 1 Nephrology, Foundation University School of Health Sciences, Rawalpindi, PAK; 2 Biochemistry, Islamic International Medical College, Rawalpindi, PAK; 3 Biochemistry, Mohi-ud-Din Islamic Medical College AJK, Mirpur, PAK; 4 Nephrology, Pakistan Institute of Medical Sciences, Islamabad, PAK; 5 Medicine/Endocrinology, Capital Development Authority (CDA) Hospital, Islamabad, PAK; 6 General Surgery, Combined Military Hospital, Muzaffarabad, PAK; 7 Surgery, District Headquarter Hospital, Muzaffarabad, PAK; 8 Pathology, Abbas Institute of Medical Sciences, Muzaffarabad, PAK

**Keywords:** diabetic, glomeruli, kidney, nephropathy, nitrogenous waste, renal

## Abstract

Introduction: Diabetes mellitus is a common metabolic disorder that is sometimes responsible for kidney diseases, especially in the form of diabetic nephropathy. In this study, we tried to investigate the markers associated with kidney diseases within diabetic patients as compared to non-diabetic participants.

Methodology: In this study, among 237 participants, 81 patients were diabetic, whereas 156 were non-diabetic participants. The level and association of serum creatinine, blood urea nitrogen (BUN), and glomerular filtration rate (GFR) were investigated using the enzymatic method and CKD-EPI equation respectively.

Result: We found significantly higher creatinine and BUN levels and lower GFR in the diabetic group compared to the non-diabetic group. Besides, we determined a positive association between creatinine and BUN, and an inverse relationship between GFR and creatinine and BUN respectively which was highly scattered in the case as compared to the non-diabetic group. Further analysis of the participants with high creatinine levels only supported the main outcomes.

Conclusion: Our investigation of kidney markers suggests a significant association between diabetes and kidney complications. Leading a healthy lifestyle and maintaining blood sugar and pressure may help to slow down the progress of diabetic nephropathy for diabetic patients.

## Introduction

Diabetes mellitus (DM) is a metabolic disorder, especially limiting carbohydrate metabolism, resulting in underusing glucose and producing hyperglycemia or blood sugar [1]. Research says the prevalence of DM will rise from 6.4% (approximately 285 million people) in 2010 to 7.7% by the end of 2030 with approximately 438 million diseased people. This percentage may increase up to 8% in the 21st century [2]. It is responsible for diabetic nephropathy and can cause renal complications which ultimately lead patients to mortality and morbidity [3].

Diabetic nephropathy is a type of kidney disease that usually occurs in diabetic patients suffering from diabetes for long periods or those whose diabetes is not under control [4]. As a result, gradually the filtering organelles inside the kidney get ruptured, and the macro protein molecules start to leak and are lost through urination [5]. To observe the stability of kidney function different tests are done and among them, creatinine, blood urea nitrogen (BUN), and glomerular filtration rate (GFR) are some of the well-recognized [6-8]. Creatinine is recognized as one of the uncharged small molecules (113 Da) and endogenous substances that is produced through the non-enzymatic transformation of phosphate and creatine molecules that remain within the serum until filtered by the glomerulus of the kidney and excreted through urination [9]. BUN, on the other hand, is a nitrogenous protein molecule regarded as metabolic waste. It is mainly produced by the liver after metabolism and filtered by the kidney [10]. Again, the GFR is a method regarded as the exogenous filtration marker [11]. Previously creatinine has been suggested as a crucial indicator of renal failure in kidney complications. However, an enhanced BUN level was regarded as an indicator of interrupted protein metabolism in renal diseases [12]. The GFR level on the other hand was considered as a critical measure for acute and chronic kidney diseases [13]. Although these markers are normally tested and observed in kidney patients, diabetic patients who have developed kidney and renal complications may check their kidney function through these tests.

In this study, we primarily tried to observe the level serum of creatinine, BUN, and GFR among diabetic patients with no diagnosed kidney diseases. Besides, we tried to compare serum creatinine and BUN levels between diabetic and non-diabetic participants, assess the GFR in both groups, and investigate the associations among creatinine, BUN, and GFR specifically in diabetic patients.

## Materials and methods

Study approval and participants

The study setting for this observational study was set up in the Muzaffarabad Center, Pakistan. Ethical approval was received from the Abbas Institute of Medical Sciences, Muzaffarabad, AJK, Pakistan with approval number 5493. A total of 237 participants were included in this study including 81 as the diabetic group and 156 as the non-diabetic group. Informed consent was obtained from each participant before collecting the samples with a short questionnaire form. 

Inclusion and exclusion criteria

As per the inclusion criteria, patients with diabetes were selected as the diabetic group. In contrast, participants who came for random check-ups with no diabetes or other certain diagnosed diseases or any symptoms were regarded as the non-diabetic group. The participants of the non-diabetic control group were confirmed to have no diabetes based on their oral glucose tolerance test (OGTT) and hemoglobin A1c (HbA1c) tests. Both the male and female participants were considered eligible for this study. No age restriction was maintained. Per the exclusion criteria, participants with other confirmed chronic diseases were excluded from the diabetic and non-diabetic groups. Again, participants with confirmed diabetes were excluded from the non-diabetic group. 

Sample collection

The blood sample of the participants was collected using the venipuncture method in the early morning in fasting conditions. The samples were centrifuged after the collection and the serum was separated from the hematocrit. Serum samples were stored at -20°C for further tests. 

Measuring diabetic parameters

We initially did the OGTT for all the patients to confirm and distinguish between diabetic and non-diabetic participants. We also measured the HbA1c parameter for each participant to confirm diabetes further. 

Biochemical test

Creatinine and BUN were tested using the Abbott architect system using their specific kit. Creatinine was measured following the principle of kinetic test based on alkaline picrate [14]. On the other hand, BUN was measured following the enzymatic testing systems based on urease [15]. The final concentration measurement for the creatinine and BUN for both tests was analyzed through a spectrophotometer. According to the kit, the reference range for serum creatinine was <0.55 mg/dL (low) to >1.02 mg/dL (high) for females, and <0.73 mg/dL (low) to >1.18 mg/dL high for males. For BUN the range was <8 mg/dL (low) to >22 mg/dL (high), and between 8 and 22 mg/dL it was referred to as the normal range. 

GFR measurement

The estimated GFR (EGFR) was measured for all the diabetic cases as well as non-diabetic participants. To calculate the value of the EGFR, the CKD-EPI creatinine equation was obtained [16]. The reference range for the GFR was <60 ml/min/1.73m^2^ as low and >60 ml/min/1.73m^2^ as normal.

Participants with a higher creatinine level

We further separated the participants of the diabetic and non-diabetic groups with higher creatinine levels. We tried to compare the markers between those groups to observe the differences and significance. 

Statistical analysis

Statistical analyses were done to understand the correlation among creatinine, BUN, and GFR. The mean, SD, and one sample t-test to obtain the p-value and investigate the significance were determined using IBM SPSS Statistics for Windows, Version 16 (Released 2007; IBM Corp., Armonk, New York, United States). The mean differences of the levels between the two groups were analyzed with RevMan (version 5.4, Cochrane, London). Scatter dot plots were generated to visualize the relationship among these biomarkers using Microsoft Excel 365 (Microsoft Corporation, Redmond, USA). The associations between biomarkers were further correlated with Pearson’s correlation. 

## Results

Demographics of participants

The number of total study participants followed by the participant numbers based on gender (i.e. male and female) and percentages, as well as the mean ± standard deviation (SD), and median value of the age, age range, OGTT (fasting and after two hours), HbA1c, ethnicity, and duration of diabetes of participants were determined. The detailed demographics are elaborated in Table 1.

**Table 1:** Demographics of study participants n: number of participants; NA: not applicable; oral glucose tolerance test

Participant description	Diabetic group	Non-diabetic group
Participants (n)	81	156
Female (n)	43	65
Male (n)	38	91
Female (%)	53.09	58.33
Male (%)	46.91	41.67
Age (mean ± SD) (in year)	58.04 ± 15.69	48.86 ± 14.71
Age (median) (in year)	58.50	49
Age range (in year)	34-82	24-72
OGTT (fasting) (mg/dL)	111.05 ± 13.73	87.81 ± 12.96
OGTT (after two hours) (mg/dL)	185.79 ± 44.76	102.13 ± 60.74
HbA1c (%)	9.29 ± 7.45	5.27 ± 0.49
Ethnicity	Asian (Pakistani)	Asian (Pakistani)
Duration of diabetes	≥ 1 year	NA

Comparison of biomarker levels

**Figure 1:**
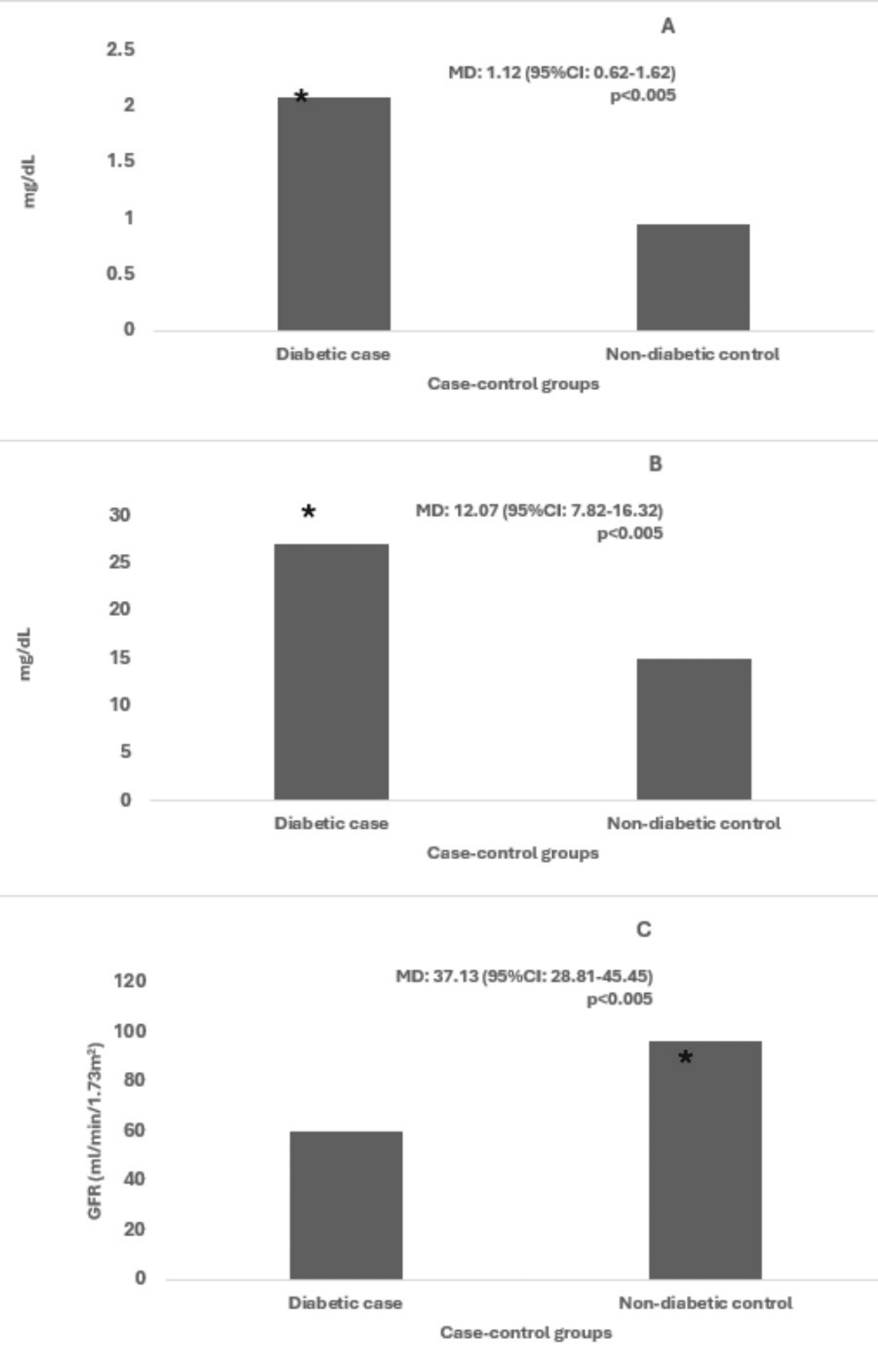
Different levels (i.e. mean value) of serum creatinine (A), BUN (B), and GFR (C) in diabetic cases and non-diabetic control MD denotes the mean differences between the levels and 95%CI denotes the 95% confidence interval. The asterisk (*) symbol denotes significantly high (p<0.005).

Comparing the levels of serum creatinine, we identified that the level was higher in diabetic cases (2.08 ± 2.26 mg/dL) as compared to non-diabetic control (0.95 ± 0.69 mg/dL) (Figure 1a). In the case of BUN, the level was also found higher in the diabetic group (27.05 ± 18.05 mg/dL) as compared to the non-diabetic control (14.98 ± 9.42 mg/dL) (Figure 1b). However, the opposite relationship was observed in the case of GFR where the level was higher in non-diabetic control (96.72 ± 23.77 ml/min/1.72m^2^) as compared to the diabetic case group (59.59 ± 34.16 ml/min/1.72m^2^) (Figure 1c). The mean differences of the levels of serum creatinine, BUN, and GFR were found to be 1.12 (95%CI: 0.62-1.62), 12.07 (95%CI: 7.82-16.32), and 37.13 (95%CI: 28.81-45.45) respectively. All the comparisons were determined to be highly significant (p<0.005) respectively (Figure 1).

Association of biomarkers

**Figure 2:**
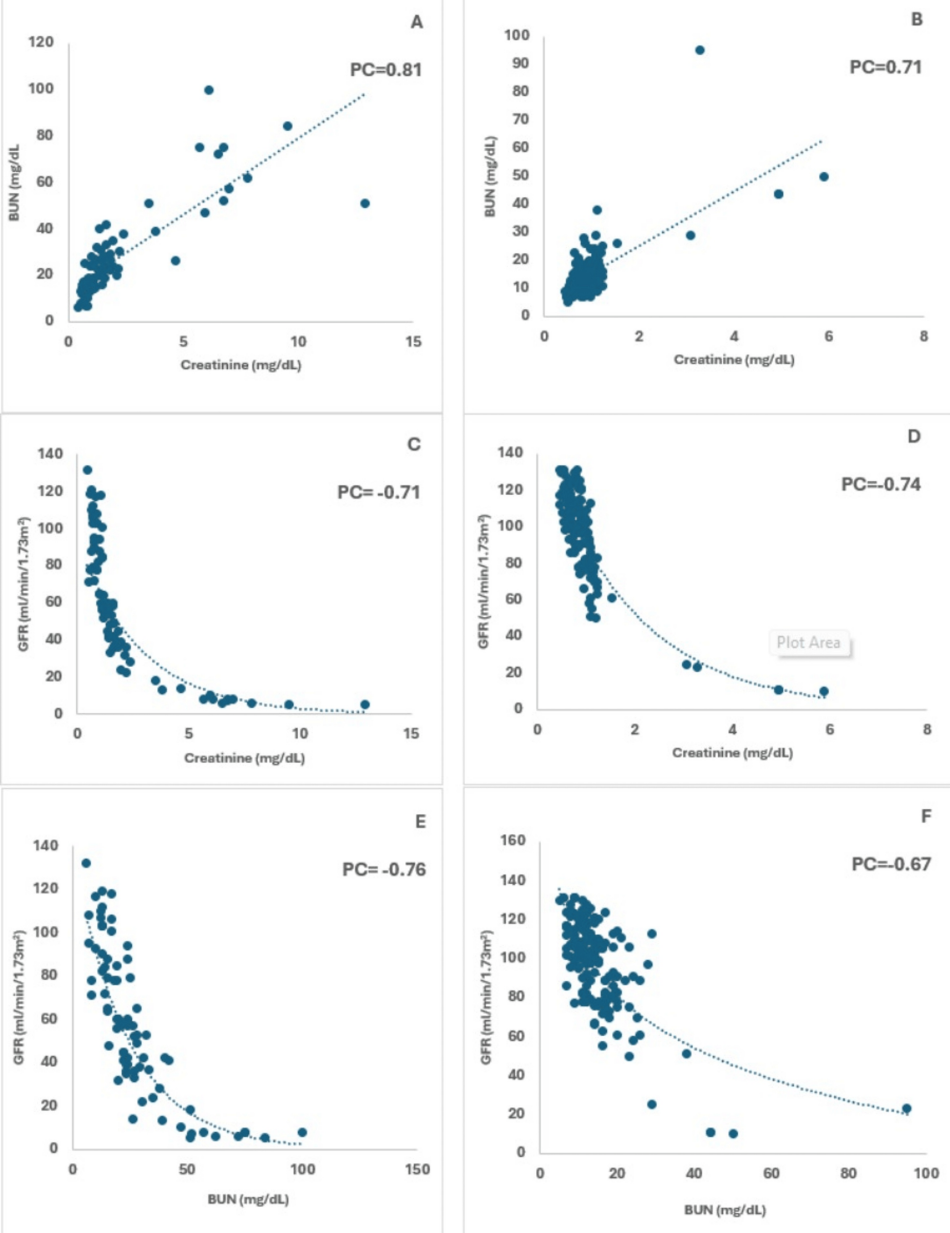
Association between creatinine and BUN Plots resemble the association between creatinine and BUN (A), creatinine and GFR (C), and BUN and GFR (E) in the diabetic group and the association between creatinine and BUN (B), creatinine and GFR (D), and BUN and GFR (F) in the non-diabetic group respectively. Here, PC indicates Pearson’s correlation.

The association of serum creatinine and BUN, serum creatinine and EGFR, and BUN and GFR between diabetic cases and non-diabetic control was investigated using scatter dot plots (Figure 2). In the diabetic group, we observed a positive but scattered correlation between creatinine and BUN and an inverse correlation between creatinine and GFR. However, a negative correlation was also observed between BUN and GFR which was highly scattered (Figures 2A, 2C, 2E). In the non-diabetic control group, the correlation was much homogenized being less scattered and similar with slight variation for creatinine and BUN (Figure 2B), creatinine and GFR (Figure 2D), and BUN and GFR (Figure 2E).

Separate analysis of participants with a risk of kidney dysfunction

We further categorized and analyzed the participants for both the diabetic and non-diabetic control groups that have higher creatinine levels as it is regarded as one of the most crucial biomarkers for possible kidney dysfunction. We determined that only 12.17% of the participants in the non-diabetic group had higher (>ref value) creatinine levels whereas approximately 58.02% of patients had higher creatinine levels in the diabetic group with a significantly high variation (p<0.005). Further analysis showed that despite high creatinine levels, the value was much lower in the non-diabetic group than in the diabetic group.

Evaluation of the BUN and GFR of both the groups with higher creatinine levels also demonstrated that the value of BUN was much lower in the non-diabetic group than in the diabetic group. The GFR on the other hand was higher in the non-diabetic group as compared to the diabetic group. All the differences were highly significant (p<0.005) (Table 2).

**Table 2:** Analysis of markers in participants that had enhanced creatinine levels (>ref value) in both diabetic and non-diabetic groups The values are expressed as mean ± standard deviation (SD), and all the comparisons were significantly different (p<0.005).

Participants and markers	Diabetic group	Non-diabetic group	p-value
Participants with enhanced creatinine (n, %)	47 (58.02%)	19 (12.17%)	
Creatinine level (mg/dL)	2.97 ± 2.62	1.98 ± 1.56	<0.005
BUN level (mg/dL)	35.32 ± 19.51	27.78 ± 19.80	<0.005
GFR (ml/min/1.73m^2^)	36.25 ± 20.94	56.58 ± 29.30	<0.005

## Discussion

Diabetic nephropathy commonly occurs in diabetic patients and more frequently in end-stage renal disease. According to statistics, approximately 30% of diabetic patients worldwide have diabetic nephropathy [17]. Besides, diabetic nephropathy has been a major portion with about one-third of the disability-adjusted life-years among all types of kidney diseases worldwide from 1997-2017. This has also been increasing yearly [18,19]. To prevent these complications, regular check-up is important for diabetes patients. 

In this study, we determined the creatinine, BUN, and GFR levels in both diabetic cases and non-diabetic control. No participants in this study were previously diagnosed or confirmed to be kidney patients. However, we saw a significant variation in the level of all these markers between diabetic and non-diabetic participants, and the level was much higher than the reference range in diabetic cases (Figure 1). Previous studies also found higher creatinine levels in diabetic patients which supports our findings. Navale et al. claimed the creatinine value to be around 1.90 mg/dL which is close to our determined value (2.08 ± 2.26 mg/dL) [20]. Another study also found higher creatinine levels in patients with type 2 DM [21]. BUN was also found to be higher in diabetic patients with different values for ages <45 years (22.43 ± 5.00 mg/dL) and >45 years (26.73 ± 6.21 mg/dL) [22]. Another study also determined higher levels of BUN in both Type 1 (27.23 ± 7.39 mg/dL) and Type 2 (28.4 ± 3.78 mg/dL) DM [23]. This data regarding BUN further supports our findings.

According to a previous study, the GFR was also investigated to be <60, mL/min/1.73m^2^ in seven diabetes patients out of 45 patients which is low compared to the regular GFR value [24]. Another study found that more than 20% of their study participants with DM had lower GFR values (<60 mL/min/1.73m^2^) and more than 32% had low to moderate range of GFR (60-75 mL/min/1.73m^2^) [25]. This also supports our observations regarding the lower GFR in diabetic patients as compared to the non-diabetic control group.

All these findings imply that diabetes patients have a higher risk of deteriorated function of the kidney which may ultimately result in kidney damage. Besides, the positive scattered (i.e. positive Pearson’s correlation) and heterogeneous association between serum creatinine and BUN also implies the plausible combined effect of kidney deterioration in diabetic patients (Figure 2). However, the opposite (i.e. negative Pearson’s correlation) and scattered correlation between GFR and creatinine as well as BUN indicates the possible destruction of kidney function that may lead the patients towards diabetic nephropathy and chronic kidney diseases (Figure 2). However, normal levels and the non-scattered association for all these markers in non-diabetic patients indicate the normal function of the kidney. Usually, creatinine is a nitrogenous waste that is excreted through the help of the kidney. BUN is another potential nitrogenous end product similar to creatinine that is produced by amino acid and protein catabolism and excreted by kidney. However, when kidney function is disrupted the level of both creatinine and BUN is enhanced in the blood [26]. Besides, the GFR is usually the conversion rate of impure blood transporting nitrogenous waste materials to ultrafiltrate blood which is performed by the kidney. Therefore, when the kidney function is disrupted, the GFR gets lower [27]. These biological phenomena further support our investigations and associations to be accurate. 

Although 12% of the participants in the non-diabetic group showed slightly higher creatinine levels, the level was still much lower than that of the diabetic group. Besides, further comparison of the BUN and GFR in these diabetic and non-diabetic groups with higher creatinine levels demonstrated better kidney function in the non-diabetic group than in the diabetic group (Table 2). The possible reason for the participants having slightly higher creatinine levels might be some kidney or renal complications other than diabetic nephropathy. Further studies need to be done to focus on these issues.

Strength of the study

This study appropriately investigated the association and variation among the creatinine, BUN, and GFR in both diabetic and non-diabetic groups. These findings would help physicians make better decisions for diabetic patients who are suffering for a long period and are at risk of developing diabetic nephropathy. This study also would enlighten physicians and clinical researchers regarding the early diagnosis, prognosis, and prevention of diabetic nephropathy and would be a food for thought for further research. 

Limitations of the study

We could not confirm whether non-diabetic participants had any other diseases or were fully healthy. We could only confirm that they did not have diabetes. Again, we did not have any confirmed diabetic nephropathy group to compare our obtained data with them. Additionally, the sample was relatively small, and the number of participants, age, and sex did not match accurately between the two groups. Besides, the study participants were of a specific location, and therefore, this study lacks generalization of the outcome. Further study regarding age-sex-matched prospective case-control research can overcome these limitations. 

## Conclusions

This study found a negative association of kidney markers in diabetic patients as compared to non-diabetic controls. From this observation, it can be said that diabetic patients have a higher risk of kidney damage through diabetic nephropathy. Maintaining a balanced life with controlled blood sugar and hypertension can minimize the chances of diabetic nephropathy. Taking no or very small amount of sugar occasionally, walking and exercising daily, and eating fiber, drinking enough water, and regular check-ups as per the advice of a physician can help to control the blood sugar and diabetes and prevent the risk of diabetic nephropathy. However, further research is required to observe the level of other associated markers to predict the possibilities of diabetic nephropathy in diabetic patients. 
